# Neutrophils in Ocular Diseases

**DOI:** 10.3390/ijms25147736

**Published:** 2024-07-15

**Authors:** Sridhar Bammidi, Victoria Koontz, Pooja Gautam, Stacey Hose, Debasish Sinha, Sayan Ghosh

**Affiliations:** 1 Department of Ophthalmology, University of Pittsburgh School of Medicine, Pittsburgh, PA 15219, USA; sri.bammidi@pitt.edu (S.B.); vik77@pitt.edu (V.K.); pog10@pitt.edu (P.G.); stacey.hose@pitt.edu (S.H.); debasish@pitt.edu (D.S.); 2 The Wilmer Eye Institute, The Johns Hopkins University School of Medicine, Baltimore, MD 21231, USA

**Keywords:** neutrophils, innate immune cell, retinal degeneration, infection, cancer, NETosis, inflammation

## Abstract

Neutrophils, traditionally viewed as first responders to infection or tissue damage, exhibit dynamic and diverse roles in ocular health and disease. This review elaborates on previous findings that showed how neutrophils contribute to ocular diseases. In ocular infections, neutrophils play a pivotal role in host defense by orchestrating inflammatory responses to combat pathogens. Furthermore, in optic nerve neuropathies and retinal degenerative diseases like age-related macular degeneration (AMD) and diabetic retinopathy (DR), neutrophils are implicated in neuroinflammation and tissue damage owing to their ability to undergo neutrophil extracellular trap formation (NETosis) and secretion of inflammatory molecules. Targeting neutrophil-dependent processes holds promise as a therapeutic strategy, offering potential avenues for intervention in ocular infections, cancers, and retinal degenerative diseases. Understanding the multifaceted roles of neutrophils in ocular diseases is crucial for developing targeted therapies to improve patient outcomes.

## 1. Introduction

Neutrophils are white blood cells, playing a fundamental role in the body’s immune response [1]. They originate from hematopoietic stem cells in the bone marrow through a process called granulopoiesis. During granulopoiesis, these stem cells differentiate into myeloblasts, promyelocytes, myelocytes, metamyelocytes, band cells, and finally, mature neutrophils [1]. Neutrophils are divided into either normal-density neutrophils (NDNs) or low-density neutrophils (LDNs). Immature neutrophils, resting neutrophils, primed neutrophils, and activated neutrophils fall under NDNs. LDNs include immature LDNs, pro-inflammatory LDNs, immunosuppressive LDNs, and exhausted neutrophils [1]. Neutrophils are short-lived; therefore, they must be constantly replenished. This intricate developmental process ensures that a constant supply of neutrophils is ready to combat infections and maintain immune homeostasis [1]. Neutrophils in mice and humans are somewhat different, with mouse neutrophils being defined by the surface expression of lymphocyte antigen 6 family member G (Ly6G) and their lack of defensins [2]. The half-life of neutrophils also differs between mice and humans; however, there is debate over the respective times. The neutrophil-to-lymphocyte ratio (NLR; an important marker for infections and disease transitions) is also different among humans (50–70%) and mice (10–30%). Furthermore, in humans, it is estimated to be around 18 h, and for mice, it is estimated to be around 6–12 h [3].

The primary function of neutrophils is to protect the body against infections, particularly bacteria and fungi [4]. They achieve this through a process called phagocytosis, wherein they engulf and destroy invading microbes [5]. The ability of neutrophils to form neutrophil extracellular traps (NETs) when activated enables efficient removal of microbes as well as debris of cells undergoing cell death [5,6]. Neutrophils also release antimicrobial molecules, such as reactive oxygen species (ROS) and antimicrobial peptides, to enhance their killing capabilities [6]. Additionally, they play a role in modulating the inflammatory response by releasing cytokines and chemokines, which recruit other immune cells to the site of infection [6].

Neutrophils are implicated in various diseases, both infectious and non-infectious. In bacterial infections, they are the first responders, rapidly migrating to the site of infection to eliminate pathogens [7]. However, dysregulated neutrophil activation can lead to tissue damage and exacerbate inflammatory conditions like rheumatoid arthritis and inflammatory bowel disease as well as several age-related and/or neurodegenerative diseases of the central nervous system (CNS) and the retina [8,9,10,11,12,13,14]. Moreover, neutrophil dysfunction is associated with certain immunodeficiencies, making individuals more susceptible to infections [15,16]. Understanding the intricacies of neutrophil development, function, and dysregulation is vital for developing targeted therapies to modulate immune responses in various disease states.

In this review, we discuss the function of neutrophils in several ocular diseases. We demonstrate that although much remains unknown, investigating the role of neutrophils in ocular diseases is warranted, particularly in ocular surface infections, optic nerve neuropathies, and retinal degenerative diseases like age-related macular degeneration (AMD) and diabetic retinopathy (DR), owing to the critical role of these immune cells in maintaining ocular health. Understanding the process of neutrophil activation could elucidate potential therapeutic targets for several ocular diseases.

## 2. Neutrophils in Corneal Infections

Neutrophils are particularly important when it comes to ocular infections, such as with a common infection, conjunctivitis, or inflammation due to keratitis. Conjunctivitis and keratitis can both be caused by viruses or bacteria within the ocular environment [17]. Neutrophils have also been shown to be detrimental in corneal infections due to herpes simplex virus type-1 (HSV-1). Neutrophils within infected corneas expressed HSV-1 antigen and lytic genes and served as a disease-causing vector when adoptively transferred into mast cell-deficient Kit(W-sh) mice, indicating that mast cells serve as protective innate immune cells, and that neutrophils are detrimental in corneal infections [18]. Neutrophils also play a significant role in the reduction and elimination of virus replication in corneal epithelial cells. [19]. NETosis is observed in the infected eyes of murine models and human cases of HSV-1 infection. Furthermore, HSV-1 infection also activates Caspase-1 activation and myeloperoxidase secretion, indicating the activation of cell death (pyroptosis) pathways by neutrophils. Additionally, neutrophil depletion accentuated ocular pathology, augmented viral load, and escalated disease scores, proving the protective role of NETs in curtailing viral replication [19]. One of the leading causes of ocular infections such as conjunctivitis is *S. aureus* [17]. Phagocytosis by neutrophils is an effective method of eliminating this bacterium, mainly through the subsequent activation of the complement system. The pathogen is detected by the surface-bound opsonin receptors on the neutrophils, such as FcγRII, FcγRIII, CR1, and CR3, subsequently leading to internalization and engulfment in a phagosome. NADPH-dependent oxidases then generate ROS to eliminate the bacteria [20]. Interestingly, if bacteria such as *S. aureus* evade phagocytosis, neutrophil extracellular traps (NETs) can be deployed to eliminate the pathogen. In addition, *S. aureus* is known to elicit a different and specific form of NETosis, a countermeasure response against the immune response, eventually allowing the bacteria to spread [21,22]. Panton–Valentine Leukocidin (PVL) is an *S. aureus*-derived bicomponent leukotoxin that partially localizes to the mitochondria and will trigger ROS and start this altered NET formation [21,22]. On the contrary, neutrophils are also targeted by *S.aureus* during infection in the eye, particularly the cornea. It is known that syndecan-1-null (*Sdc1*^−/−^) mice are resistant to *S. aureus* corneal infection compared to wild-type mice. Moreover, depletion of neutrophils in the resistant *Sdc1*^−/−^ mice increased the corneal bacterial burden, suggesting that syndecan-1 modulates neutrophil function to promote infection. Syndecan-1 significantly inhibits neutrophil-mediated bacterial killing without affecting infiltration of neutrophils into the infected cornea [23].

Other bacterial infections include inflammation of the cornea, or keratitis, which is associated with the bacterial etiological agent *Streptococcus pyogenes* and is dictated by the M-protein coat, which serves as a virulence factor [24]. However, neutrophils that detect the M-protein will release a heparin-binding protein through azurophilic granules, thus triggering the degranulation mechanism through signaling complexes that bind β1 integrins and fibrinogen, leading to bacterial elimination [24,25]. One of the key aftereffects of bacterial infections and the rise of immune cell populations in the eye is the effective resolution of the inflammatory processes. Such resolution after elimination of bacterial infections on ocular surfaces is key to re-establishing homeostasis. It has been shown that in C57BL/6 mouse corneas treated with Resolvin D1 (RvD1; anti-inflammatory molecule) after stimulation with *S. aureus*, there was reduced cytokine production in mouse corneas, corneal opacity development, thickening, and neutrophil infiltration, indicating downregulation of the inflammatory immune response [26]. Treatment with several resolving factors has been shown to be associated with effective resolution of ocular inflammation, in particular the normalization of neutrophil function after infections, and could serve as a putative therapeutic measure for such ocular diseases [27,28,29,30].

The ocular surface is very dynamic in terms of its microbial commensal population [31]. However, loss of commensal properties among these bacterial populations on the ocular surface has been associated with several infections, and the involvement of immune cells, including neutrophils, has been widely studied. In mice, loss of L-plastin (LCP1), an actin-bundling protein, resulted in an ocular commensal overgrowth, characterized by an increased presence of conjunctival *Streptococcal* spp. The commensal overgrowth correlated with susceptibility to P. aeruginosa-induced keratitis [32]. Mechanistically, the elevated commensal burden and the increase in infection susceptibility were linked to defects in neutrophil activation during homeostasis and in infection, along with a compromised bactericidal activity [32]. Additionally, using time-course transcriptome analysis, it has been noted that the neutrophil population had a sharp rise during the early stage of ocular surface infections like fungal keratitis and then gradually decreased as inflammation resolved [33]. Such activation of immune cells and subsequent immune responses led to upregulation of absent in melanoma 2 (AIM2)-, pyrin-, and Z-DNA binding protein 1 (ZBP1)-mediated PANoptosis, a newly identified cell death pathway that is regulated by multifaceted PANoptosome complexes and highlighted by crosstalk among pyroptosis (P), apoptosis (A), and/or necroptosis (N) pathways [33]. Neutrophils have also been shown to play a key role in a murine model of *P. aeruginosa* corneal infection (keratitis), where neutrophils secrete high levels of IL-1β, which requires the T3SS needle and translocon proteins along with gasdermin D (GSDMD) [34]. Both macrophages and neutrophils show effective immune response upon *P. aeruginosa* infection through inflammasome activation. Macrophages infected with PAO1 (strain of *P. aeruginosa*) and mutants lacking ExoS and ExoT (ΔexoST) require NLR family CARD domain-containing 4 (NLRC4) for IL-1β secretion [33]. Neutrophils infected with ΔexoST require NLRC4, but infection with PAO1 is dependent on Nod-like receptor family pyrin domain-containing 3 (NLRP3) and driven by the ADP ribosyl transferase (ADPRT) activity of ExoS [34]. These results indicate that diverse immune response signaling is prevalent in neutrophils and macrophages during *P. aeruginosa*-mediated corneal infections. Furthermore, ExoS ADPRT is critical in regulating inflammasome subtype usage in neutrophils versus macrophages and plays an unexpected role for NLRP3 in *P. aeruginosa* keratitis [34]. Furthermore, in *A. fumigatus* corneal infection, neutrophils are the major source of acidic mammalian chitinase (AMCase), and treatment with AMCase inhibitors or adoptive transfer of neutrophils from AMCase^−/−^ mice resulted in retarded fungal elimination. These findings suggest that chitin synthases are critical fungal virulence factors, and that neutrophil-derived AMCase is an essential regulator of host defense [35]. A mouse model of a *P. aeruginosa* corneal infection showed that treatment with anti-chemokine (C-C motif) ligand 2 (CCL2) or anti-chemokine (C-C motif) ligand 3 (CCL3) antibodies resulted in a significant reduction in the severity of corneal damage and polymorphonuclear neutrophil (PMN) infiltration at 1 and 7 days after infection, without affecting the rate of bacterial clearance from the cornea. CCL2 and CCL3 are critical regulators of PMN recruitment and may lead to therapeutic strategies for the management of *P. aeruginosa* keratitis [36]. Interestingly, neutrophil activation and NETosis formation is also known to play a dual role in the pathogenesis of *P. aeruginosa* keratitis. In a mouse model of *P. aeruginosa* keratitis, treatment with tobramycin/dexamethasone (TobraDex), 0.3% tobramycin (Tobrex), or 0.1% dexamethasone showed that Tobrex reduced neutrophil infiltration and corneal *P. aeruginosa* burden [37]. Dexamethasone reduced NETs, bacterial burden, and severe neutrophil infiltration, and TobraDex produced a greater reduction in the amount of neutrophils, NETs, and bacterial burden [37]. This was associated with clinical findings showing that TobraDex- and Tobrex-treated mice exhibited slight corneal damage, whereas dexamethasone-treated mice exhibited very severe corneal damage. These results indicate that NETosis may play a dual role of infection control and corneal damage in *P. aeruginosa* keratitis [37]. Several neutrophil-derived or neutrophil-regulating factors have been shown to be critical in fungal keratitis and other ocular infections (bacterial and viral), providing ample evidence of the importance of these neutrophils in ocular surface infections [38,39,40].

The role of neutrophils as an acute-phase immune regulator provides a potential therapeutic target for several of these ocular surface infections. Secreted factors from the neutrophils are helpful in eliminating the pathogen; however, an unregulated immune response could be menacing to the ocular tissue. Therefore, effective resolution of inflammation and clearance of immune factors and cells from the infection site is warranted to maintain homeostasis, and treatment modalities targeting these processes should be of importance.

## 3. Neutrophils in Ocular Malignancies

In the context of cancer, persistent inflammation triggered by chronic infections, autoimmune diseases, or environmental factors creates a microenvironment conducive to tumorigenesis. This inflammatory tumor microenvironment (TME) is characterized by elevated levels of acellular components such as proinflammatory cytokines, growth factors, and ROS, all of which contribute to DNA damage, increased cell proliferation, and suppression of cell death—critical hallmarks of cancer development [41,42].

In addition to acellular components, TME is also the site of different cells, tumor-associated neutrophils (TANs) and macrophages (TAMs), T-cells, B-cells, myeloid-derived suppressor cells, mast cells, granulocytes, dendritic cells, adipocytes, vascular endothelial cells, and pericytes [43]. TME formation has also been observed in the case of ocular cancers such as uveal melanoma and conjunctival melanoma [44,45]. Though direct evidence linking neutrophils to ocular cancer remains limited, the established role of neutrophils in TME formation and cancer pathogenesis across various cancer types [46,47,48,49], suggests their potential involvement in ocular malignancies. 

## 4. Neutrophils in Optic Nerve Diseases

Optic nerve diseases or optic neuropathies (ON) result from various causes including inflammation, glaucoma, ischemia, toxins/nutrition, trauma, or genetic factors. These conditions lead to damage to the optic nerve, resulting in the loss of retinal ganglion cells (RGCs), leading to irreversible visual impairment due to the limited capacity of RGC regeneration [50].

Inflammatory optic neuropathies like optic neuritis manifest as subacute unilateral vision loss, eye pain, decreased contrast/color sensitivity, and relative afferent pupillary defect [51]. The primary causes for such neuropathies are demyelination (e.g., multiple sclerosis-associated optic neuritis) and immune dysregulation. Inflammatory demyelination and pro-inflammatory cytokines contribute to conduction block in multiple sclerosis-associated optic neuritis, and neuromyelitis involves aquaporin-4 autoantibody-mediated astrocyte–microglia interaction and complement activation [51]. In the cases of auto-immune or infectious neuropathies, the visual acuity is inversely proportional to the number of infiltrating neutrophils crossing the blood–retina barrier (BRB). In the case of infectious ON, neutrophils release NETs. In addition to capturing and eliminating pathogens, NETs also aggravate the inflammatory response and thus act as a double-edged sword [52]. In ON neuropathies, the number and activation level of neutrophils in the periphery have been shown to be effective markers for assessing disease state and severity. Primary open-angle glaucoma (POAG), a classic example of optic neuropathy, exhibits slow, progressive, irreversible RGC degeneration, visual field defects, optic nerve head cupping, and elevated intraocular pressure [53]. An increased neutrophil-to-lymphocyte ratio (NLR) was observed in patients compared to controls; this ratio was directly correlated with disease severity [54]. These results suggest that the NLR levels may serve as an inflammatory predictor in POAG patients [54]. Ischemic optic neuropathy (ION) results from optic nerve infarction, classified as anterior (AION) or posterior (PION), arteritic or non-arteritic. Non-arteritic AION involves small-vessel ischemia/hypoperfusion, optic nerve swelling, compartment syndrome, and a cycle of axonal degeneration/RGC apoptosis. Arteritic AION is often caused by giant cell arteritis vasculitis [55]. NLR has also been shown to be upregulated in non-arteritic anterior ischemic optic neuropathy (NAION) and is suggestive of a new inflammatory marker for assessment of the severity of inflammation in NAION patients [56,57]. 

Neutrophils secrete a plethora of soluble factors. Dysregulation in the release of pro-inflammatory cytokines amplifies the inflammatory cascade, potentially leading to neuronal death in the optic nerve [58]. Neutrophils generate antimicrobial peptides, such as defensins and cathelicidins, which aid in combating potential bacterial or viral infections that may exacerbate optic nerve damage [59]. On the other hand, they also generate ROS through the respiratory burst, a potent antimicrobial mechanism. This excessive ROS production can induce oxidative stress, leading to lipid peroxidation, protein modification, and DNA damage, ultimately contributing to neuronal injury and demyelination [60]. In response to inflammatory stimuli, neutrophils release NETs, comprised of decondensed chromatin and antimicrobial proteins. Though NETs are initially a defensive mechanism, they can also cause collateral tissue damage and exacerbate inflammation in optic nerve diseases [61]. Neutrophils secrete proteolytic enzymes, including matrix metalloproteinases (MMPs) and neutrophil elastase, which can degrade and clear debris from the site of injury, facilitating tissue remodeling and regeneration. Experiments have shown that numerous neutrophils enter the mouse eye within a few hours of induction of an inflammatory reaction and express high levels of the atypical growth factor oncomodulin, which promotes optic nerve regeneration [62].

Neutrophils exhibit a complex duality in optic neuropathies, serving both protective and detrimental roles [50,51,52,53,54,55,56,57]. Their antimicrobial defense, debris clearance, and promotion of other immune cell recruitment contribute to inflammation resolution and tissue repair [52]. However, excessive neutrophil activation and infiltration can exacerbate damage through the release of pro-inflammatory cytokines, reactive oxygen species, proteolytic enzymes, and NETs, amplifying inflammation and compromising neuronal function [58,59,60,61,62]. This delicate balance is influenced by disease etiology, stage, and inflammatory milieu, necessitating careful modulation of neutrophil functions to harness their beneficial roles while mitigating detrimental effects for effective therapeutic interventions.

## 5. Neutrophils in Retinal Degeneration

Neutrophils are now thought to play critical roles in several retinal degenerative diseases, including AMD and DR. However, very little is still known about neutrophil regulation during these pathologies, with more emphasis placed on well-studied immune regulators and cells like microglia, macrophages/monocytes, and mast cells [63,64,65,66,67]. Herein, we aim to elucidate the function of neutrophils in retinal degenerative processes and to provide insight into whether targeting neutrophil activation or neutrophil-derived processes could be beneficial in delaying the progression of these diseases that have limited or no treatment options at present.

Neutrophils have been thought to be important in inherited retinal degenerative diseases including Stargardt disease, a rare genetic disease that causes degenerative changes in the macular region of the retina, an area that is required for visual acuity [68]. Neutrophil myeloperoxidase (MPO) triggers the H_2_O_2_-dependent oxidation of chloride anion to generate hypochlorous acid, a potent antimicrobial agent. Aberrant degranulation of neutrophils leads to the release of MPO to the extracellular space, thereby causing tissue damage through induction of oxidation of several additional substrates. RPE cells take up MPO through mannose 6-phosphate receptor and then target it to the lysosomes [69], where MPO exerts both cell-protective and cytotoxic functions. Interestingly, MPO catalyzes the in vitro degradation of *N*-retinylidene-*N*-retinylethanolamine, a toxic form of retinal lipofuscin that accumulates in RPE lysosomes and drives the pathogenesis of Stargardt macular degeneration [69]. Furthermore, chronic cellular uptake and accumulation of MPO in lysosomes reduces lysosomal pH, thereby activating transcription factor EB (TFEB) and cell death pathways. These results indicate that acute exposure of neutrophil degranulation proteins to RPE cells triggers the elimination of harmful lipofuscin components, whereas chronic exposure results in progressive accumulation of MPO in lysosomes leading to cell death and might be associated with the progression of Stargardt disease [69].

Neutrophils are now thought to be critical for disease progression in AMD. It has been shown that patients with AMD have a higher NLR compared to healthy controls [70]. Moreover, it has been shown that NLR correlates with disease severity and may serve as a biomarker of inflammation in AMD [71]. Additionally, patients with neo-vascular AMD showed increased expression of the activity marker cluster of differentiation (CD) 66b, decreased expression of adhesion marker CD162, and diminished expression of the resolution of inflammation marker C-X-C chemokine receptor 2 in peripheral blood, suggesting that the activity of circulating neutrophils may differ in patients with neovascular AMD [72]. These results provide evidence that neutrophils are altered in number and activation level in human AMD patients, which could possibly serve as a marker for disease onset. However, larger cohorts need to be evaluated to further establish these findings.

Studies on animal models and human AMD donor tissues suggested that neutrophils infiltrate the retina in non-exudative/dry AMD and trigger retinal degeneration [10,11,12]. Using mouse models that lack the *Cryba1* gene (which encodes βA3/A1-crystallin, a lysosomal luminal protein) and *Akt2* knockin (KI) specifically in the RPE (*Cryba1* cKO and *Akt2* KI) and develop an AMD-like phenotype with age, it was shown that neutrophils infiltrate the sub-retinal space of these mouse models with age [10,11,73,74]. Additionally, in a separate well-studied animal model of wet AMD, there was also retinal infiltration of neutrophils [75]. IL-1β (a major inflammatory cytokine associated with AMD) was localized to Ly6G-positive, Iba1-negative infiltrating neutrophils in laser-induced choroidal neovascularization (CNV) lesions in the retina [75]. In addition to these mouse models, human dry AMD cadaver sections also showed extensive infiltration of activated neutrophils as evident from the expression of neutrophil degranulation proteins Lipocalin-2 and MPO as well as NETosis markers, and citrunilated histone positivity in these cells [11,12], suggesting that the infiltration and degranulation of activated neutrophils could thereby be responsible for tissue damage in the retina, leading to progression of AMD. 

**Figure 1:**
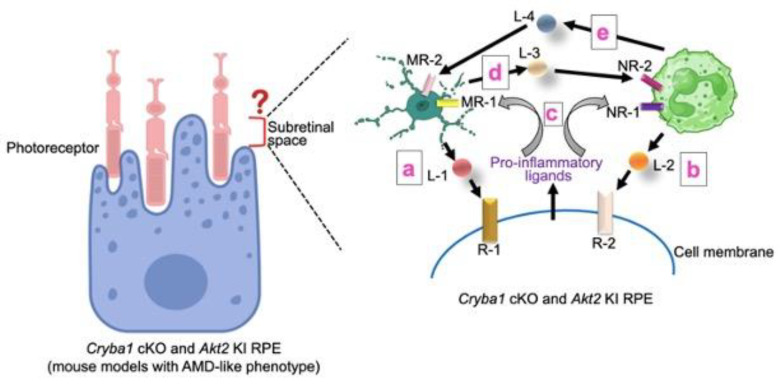
Dynamic interactions in the subretinal space during AMD progression. Multiple interactions can occur at the subretinal space during AMD progression. Ligand–receptor (LR) pairs interact in cell types known to be critical for AMD pathogenesis like the RPE, microglia, and neutrophils. Understanding these cell–cell interactions, including those between (a) microglia and RPE, (b) neutrophils and RPE, (c) RPE and microglia and/or neutrophils, (d) microglia and neutrophils, and (e) neutrophils and microglia, could be beneficial in understanding the pathways associated with chronic inflammatory transitions, known to be critical in AMD progression. L-1 to 4: Ligand 1 to 4; R-1 and 2: RPE receptor 1 and 2; MR-1 and 2: microglia receptor 1 and 2; NR-1 and 2: neutrophil receptor 1 and 2. Template for RPE, neutrophils, and microglia was obtained from BioRender.com accessed on 26 June 2024.

RPE cells govern the anti-inflammatory niche in the sub-retinal space (SRS), a region now thought to be critical for the chronic inflammation associated with AMD [10,11,12,63,64,65]. However, diseased RPE cells trigger infiltration of several immune cells into the sub-retinal space and facilitate their persistence, which subsequently leads to retinal degenerative changes [64,65]. Additionally, other immune cells, like microglia (resident immune cells in the retina), also infiltrate the SRS in the disease state [63]. Microglia have been shown to play a critical dual role in both homeostatic and degenerative processes in the retina [10,63]. The question now is whether there is any dynamic interaction of these immune cells (microglia, neutrophils) in the SRS and what the role of the RPE is in this context (Figure 1). It is known that microglia can eliminate infiltrating neutrophils [76]. However, it can be speculated that in AMD, the changes in the immune niche within the SRS, alterations in RPE function, and the release of pro-inflammatory mediators could reduce the immune surveillance function of the microglia (Figure 1). It has been shown by extensive scRNAseq analysis followed by bioinformatics using the ligand–receptor (LR) loop tool that each of these cell types (microglia, neutrophils, and RPE) shows dynamic interactions in the SRS of a mouse model as the disease progresses [10]. Additionally, RPE-derived factors trigger the activation of microglia, which subsequently activate neutrophils, leading to NETosis formation and LCN-2 and MPO activation [10]. These activated neutrophils could also induce early RPE changes in immune-compromised mice [10]. Therefore, on the basis of these findings, it is understandable that targeting these pro-inflammatory processes in the RPE could be beneficial by inhibiting the infiltration of neutrophils and other immune cells into the SRS. It has been shown that inhibiting AKT2 in the RPE of *Cryba1* cKO and *Akt2* KI mice diminishes abnormal microglial activation, neutrophil infiltration, and early RPE changes in mouse models [10,11]. Targeting these processes is critical and could have therapeutic importance, leading to the development of therapeutic interventions to delay the progression of AMD. However, in-depth analysis must be explored to identify specific cell–cell interaction pathways at the SRS, particularly using both mouse models and human AMD donor tissues.

In addition to AMD, neutrophils have also been shown to play a critical role in DR [13,14]. Studying retinal tissues from human donors with proliferative diabetic retinopathy and a mouse model of ischemic retinopathies, researchers uncovered a new role for neutrophils in vascular remodeling during disease pathogenesis [77]. They found that senescent blood vessels release signals that attract neutrophils and induce the production of NETs, which ultimately clear diseased endothelial cells and remodel unhealthy vessels. Inhibiting NETosis prevented the regression of senescent vessels and prolonged disease, indicating that clearing senescent retinal blood vessels promotes reparative vascular remodeling [77]. Furthermore, neutrophil elastase (NE), a serine protease secreted by neutrophils, is known to be elevated in diabetes [13]. There is an increase in retinal vascular permeability and levels of NE in both the retina and plasma of diabetic wild-type animals. These abnormalities are significantly reduced in mice lacking elastase (Elane^−/−^) [13,14]. In vitro studies show that NE increases retinal endothelial cell permeability, partly inhibited by MyD88, NF-κB, and PAR2 inhibitors, and that NE degrades VE-cadherin in a concentration-dependent manner [13]. In mice diabetic for 2 months, deletion or selective inhibition of NE reduces diabetes-induced retinal superoxide levels and inflammation and prevents leukocyte-mediated cytotoxicity of retinal endothelial cells. In mice diabetic for 8 months, genetic deletion of NE significantly inhibits diabetes-induced retinal capillary degeneration [13,14]. These results suggest that proteases released from neutrophils contribute to the development of DR, and that targeting these proteases could be a novel therapy for DR [13,14]. 

It is to be noted that in DR, neutrophils have differential functions both as a protective and as an adverse entity. It has been shown that neutrophils can serve as a protective mediator by eliminating senescent cells when the disease has been established, as evident from the studies on human donor tissues with DR [77]. However, these innate immune cells play a detrimental role during the development of DR, as evident from the animal studies on *Elane*^−/−^ mice [13,14]. Therefore, extensive research is warranted to pinpoint specific pathways by time course studies to understand the extent of neutrophil activation during DR progression. 

## 6. Discussion

Clearly, neutrophils have multifaceted roles in various ocular diseases, including infection, malignancies, and optic nerve and retinal degenerative diseases. In ocular surface infections, neutrophils act as acute-phase immune regulators, releasing factors to help eliminate pathogens [21,22,23,24,25]. However, unregulated neutrophils may cause detrimental consequences to the eye’s surface. Treatment with several anti-inflammatory agents has been shown to be an effective avenue for maintaining homeostasis in infections of the ocular surface [26,27,28,29,30]. 

Neutrophils have also been shown to have both protective and detrimental roles in optic nerve generation and neuropathies. The protective roles encompass the anti-microbial defense [60,62]; this homeostatic immune cell activation is overpowered when unregulated neutrophil activation due to genetic predisposition and/or environmental factors leads to harmful effects such as chronic inflammation and compromised neuronal function through the formation of NETs [59,61]. It should be noted that the underlying signaling associated with this differential regulation of neutrophil function remains elusive. Therefore, pinpointing the factors and the molecular pathways that drive altered activation of neutrophils could be beneficial in managing diseases involving optic nerve degeneration.

In diseases like AMD and DR, the leading cause of blindness in the modern world, inflammation plays a key role, and neutrophils have shown to be associated with the pathogenesis of these diseases [10,11,12,13,14,77,78,79,80]. They infiltrate the retina, leading to inflammation and neuronal damage [10,11,12,13,14]. Though other immune cells have been shown to be important for AMD progression [63,64,65,66,67], very little is known about how neutrophils infiltrate the retina in AMD and cause retinal degenerative changes. In AMD, the loss of the RPE’s anti-inflammatory functions and the subsequent release of pro-inflammatory mediators is thought to trigger neutrophil infiltration into the SRS and activation [10,11,12]. In the disease state, the dynamic cell–cell interactions in the SRS (between the diseased RPE, microglia, and infiltrating neutrophils) are now thought to be critical for the onset of retinal degeneration, as seen in AMD [10]. Therefore, understanding these cellular interactions could be important in establishing treatment modalities to inhibit the activation of chronic inflammation, a critical process in AMD pathogenesis. Interestingly, in DR, neutrophils have protective functions, such as eliminating senescent cells and maintaining retinal homeostasis [76], but they also contribute to both disease onset and progression [13,14]. Understanding this stage- and context-dependent function of neutrophils is essential for developing targeted therapeutic strategies for these debilitating retinal degenerative diseases. 

## 7. Conclusions

**Figure 2:**
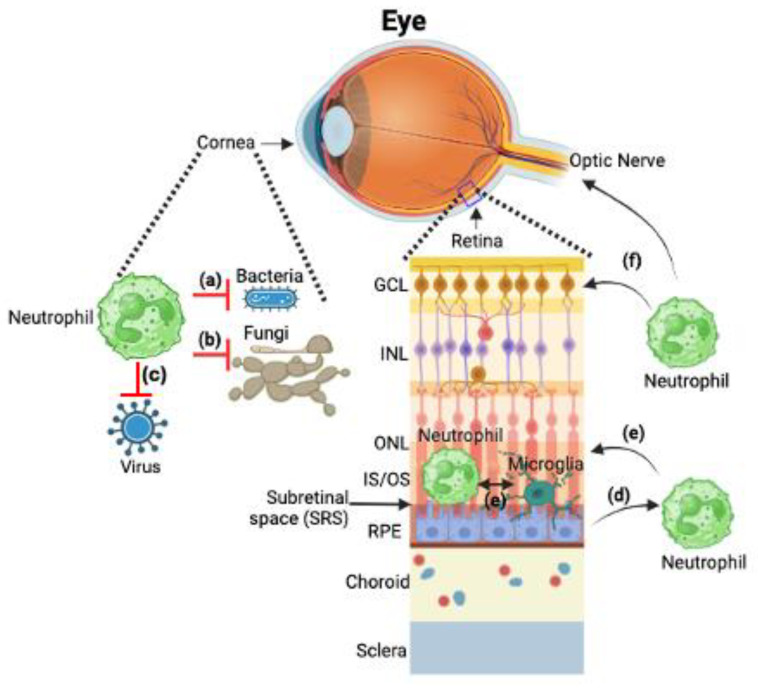
Neutrophils in ocular pathologies. Eye anatomy showing the cornea and retina as well as the optic nerve and retinal vasculatures (top panel). Neutrophils play a critical role in elimination of (a) bacteria, (b) fungi, and (c) virus infections on ocular surfaces, particularly the cornea. In dry AMD, (d) diseased RPE releases soluble factors that drive neutrophil activation and infiltration into the SRS. (e) Subsequently, these infiltrating neutrophils can interact with activated resident immune cells (microglia) at the SRS, leading to retinal degeneration. (f) In DR and optic nerve neuropathies, neutrophils show both detrimental and protective roles through remodeling of the retinal vasculature and protective mechanisms on RGCs at the ganglion cell layer (GCL) and inner and outer plexiform layers (IPL/OPL) layers with inner retinal blood vessels. Therefore, targeting processes that effect neutrophil function during disease progression could provide an interesting therapeutic target for these ocular diseases. The figure was created by BioRender.com accessed on 26 June 2024.

In conclusion, it can be stated that neutrophils are central to the pathophysiology of various ocular diseases, exerting diverse effects ranging from protective to detrimental (Figure 2). Further research into their specific functions in different disease contexts is crucial for developing effective therapeutic interventions.

## 8. Future Directions

In-depth experimental validations using both human donor samples and mouse models is required to identify specific signaling molecules (like pro-inflammatory mediators and NETosis components) that govern neutrophil activation in ocular pathologies. It is understandable that there is a scarcity of human donor tissues; therefore, in vitro iPSC-derived models (i.e., disease in a dish) from human donors could potentially serve as an effective alternative to understand the underlying signaling, and they could be an important tool for putative drug screening.

## References

[1] Hong C.W. (2017). Current Understanding in Neutrophil Differentiation and Heterogeneity. Immune Netw..

[2] Hackert N.S., Radtke F.A., Exner T., Lorenz H.M., Müller-Tidow C., Nigrovic P.A., Wabnitz G., Grieshaber-Bouyer R. (2023). Human and mouse neutrophils share core transcriptional programs in both homeostatic and inflamed contexts. Nat. Commun..

[3] Hidalgo A., Casanova-Acebes M. (2021). Dimensions of neutrophil life and fate. Semin. Immunol..

[4] Rasmussen T.P., Echarri Á.R., Cox J.D., de Abajo F.J.G. (2024). Generation of entangled waveguided photon pairs by free electrons. Sci. Adv..

[5] Park J., Wysocki R.W., Amoozgar Z., Maiorino L., Fein M.R., Jorns J., Schott A.F., Kinugasa-Katayama Y., Lee Y., Won N.H. (2016). Cancer cells induce metastasis-supporting neutrophil extracellular DNA traps. Sci. Transl. Med..

[6] Selders G.S., Fetz A.E., Radic M.Z., Bowlin G.L. (2017). An overview of the role of neutrophils in innate immunity, inflammation and host-biomaterial integration. Regen. Biomater..

[7] Fine N., Tasevski N., McCulloch C.A., Tenenbaum H.C., Glogauer M. (2020). The Neutrophil: Constant Defender and First Responder. Front. Immunol..

[8] Fresneda Alarcon M., McLaren Z., Wright H.L. (2021). Neutrophils in the Pathogenesis of Rheumatoid Arthritis and Systemic Lupus Erythematosus: Same Foe Different M.O. Front. Immunol..

[9] Zenaro E., Pietronigro E., Della Bianca V., Piacentino G., Marongiu L., Budui S., Turano E., Rossi B., Angiari S., Dusi S. (2015). Neutrophils promote Alzheimer’s disease-like pathology and cognitive decline via LFA-1 integrin. Nat. Med..

[10] Boyce M., Xin Y., Chowdhury O., Shang P., Liu H., Koontz V., Strizhakova A., Nemani M., Hose S., Zigler J.S. (2022). Microglia-Neutrophil Interactions Drive Dry AMD-like Pathology in a Mouse Model. Cells.

[11] Ghosh S., Padmanabhan A., Vaidya T., Watson A.M., Bhutto I.A., Hose S., Shang P., Stepicheva N., Yazdankhah M., Weiss J. (2019). Neutrophils homing into the retina trigger pathology in early age-related macular degeneration. Commun. Biol..

[12] Ghosh S., Shang P., Yazdankhah M., Bhutto I., Hose S., Montezuma S.R., Luo T., Chattopadhyay S., Qian J., Lutty G.A. (2017). Activating the AKT2-nuclear factor-κB-lipocalin-2 axis elicits an inflammatory response in age-related macular degeneration. J. Pathol..

[13] Lessieur E.M., Liu H., Saadane A., Du Y., Tang J., Kiser J., Kern T.S. (2021). Neutrophil-Derived Proteases Contribute to the Pathogenesis of Early Diabetic Retinopathy. Investig. Ophthalmol. Vis. Sci..

[14] Liu H., Lessieur E.M., Saadane A., Lindstrom S.I., Taylor P.R., Kern T.S. (2019). Neutrophil elastase contributes to the pathological vascular permeability characteristic of diabetic retinopathy. Diabetologia.

[15] Dinauer M.C. (2016). Primary immune deficiencies with defects in neutrophil function. Hematol. Am. Soc. Hematol. Educ. Program.

[16] Keszei M., Westerberg L.S. (2014). Congenital defects in neutrophil dynamics. J. Immunol. Res..

[17] Livingston E.T., Mursalin M.H., Callegan M.C. (2019). A Pyrrhic Victory: The PMN Response to Ocular Bacterial Infections. Microorganisms.

[18] Royer D.J., Zheng M., Conrady C.D., Carr D.J. (2015). Granulocytes in Ocular HSV-1 Infection: Opposing Roles of Mast Cells and Neutrophils. Investig. Ophthalmol. Vis. Sci..

[19] Patil C.D., Borase H., Gagan S., Sharma P., Kapoor D., Yadavalli T., Jain S., Joseph J., Bagga B., Shukla D. (2024). Rapid NETosis Is an Effector Mechanism to Combat Ocular Herpes Infection. Investig. Ophthalmol. Vis. Sci..

[20] van Kessel K.P., Bestebroer J., van Strijp J.A. (2014). Neutrophil-Mediated Phagocytosis of *Staphylococcus aureus*. Front. Immunol..

[21] Mazzoleni V., Zimmermann K., Smirnova A., Tarassov I., Prévost G. (2021). *Staphylococcus aureus* Panton-Valentine Leukocidin triggers an alternative NETosis process targeting mitochondria. FASEB J..

[22] Speziale P., Pietrocola G. (2021). *Staphylococcus aureus* induces neutrophil extracellular traps (NETs) and neutralizes their bactericidal potential. Comput. Struct. Biotechnol. J..

[23] Hayashida A., Amano S., Park P.W. (2011). Syndecan-1 promotes *Staphylococcus aureus* corneal infection by counteracting neutrophil-mediated host defense. J. Biol. Chem..

[24] Eichelberger K.R., Goldman W.E. (2020). Manipulating neutrophil degranulation as a bacterial virulence strategy. PLoS Pathog..

[25] Marquart M.E., O’Callaghan R.J. (2013). Infectious keratitis: Secreted bacterial proteins that mediate corneal damage. J. Ophthalmol..

[26] Kim S.Y., Lee J.E. (2023). Resolvin D1 Inhibits Corneal Inflammation in *Staphylococcus aureus* Keratitis. Ocul. Immunol. Inflamm..

[27] Rajasagi N.K., Reddy P.B., Suryawanshi A., Mulik S., Gjorstrup P., Rouse B.T. (2011). Controlling herpes simplex virus-induced ocular inflammatory lesions with the lipid-derived mediator resolvin E1. J. Immunol..

[28] Qin Q., Hu K., He Z., Chen F., Zhang W., Liu Y., Xie Z. (2022). Resolvin D1 protects against Aspergillus fumigatus keratitis in diabetes by blocking the MAPK-NF-κB pathway. Exp. Eye Res..

[29] Rajasagi N.K., Bhela S., Varanasi S.K., Rouse B.T. (2017). Frontline Science: Aspirin-triggered resolvin D1 controls herpes simplex virus-induced corneal immunopathology. J. Leukoc. Biol..

[30] Lee S., Kim S., Park S., Lee J., Yu H.S. (2022). Effect of resolvin D1 on experimental bacterial keratitis to prevent corneal scar. Graefe’s Arch. Clin. Exp. Ophthalmol..

[31] St Leger A.J., Caspi R.R. (2018). Visions of Eye Commensals: The Known and the Unknown About How the Microbiome Affects Eye Disease. Bioessays.

[32] Lu X., Kugadas A., Smith-Page K., Lamb J., Lin T., Ru Y., Morley S.C., Fichorova R., Mittal S.K., Chauhan S.K. (2020). Neutrophil L-Plastin Controls Ocular Paucibacteriality and Susceptibility to Keratitis. Front. Immunol..

[33] Xu X., Wei Y., Pang J., Wei Z., Wang L., Chen Q., Wang Z., Zhang Y., Chen K., Peng Y. (2023). Time-Course Transcriptomic Analysis Reveals the Crucial Roles of PANoptosis in Fungal Keratitis. Investig. Ophthalmol. Vis. Sci..

[34] Minns M.S., Liboro K., Lima T.S., Abbondante S., Miller B.A., Marshall M.E., Tran Chau J., Roistacher A., Rietsch A., Dubyak G.R. (2023). NLRP3 selectively drives IL-1β secretion by *Pseudomonas aeruginosa* infected neutrophils and regulates corneal disease severity. Nat. Commun..

[35] de Jesus Carrion S., Abbondante S., Clark H.L., Marshall M.E., Mouyna I., Beauvais A., Sun Y., Taylor P.R., Leal S.M., Armstrong B. (2019). Aspergillus fumigatus corneal infection is regulated by chitin synthases and by neutrophil-derived acidic mammalian chitinase. Eur. J. Immunol..

[36] Xue M.L., Thakur A., Cole N., Lloyd A., Stapleton F., Wakefield D., Willcox M.D. (2007). A critical role for CCL2 and CCL3 chemokines in the regulation of polymorphonuclear neutrophils recruitment during corneal infection in mice. Immunol. Cell Biol..

[37] Zhu B., Zhang L., Yuan K., Huang X., Hu R., Jin X. (2021). Neutrophil extracellular traps may have a dual role in *Pseudomonas aeruginosa* keratitis. Eur. J. Clin. Microbiol. Infect. Dis..

[38] Xiao Y., Yang J., Fu Z., Xiong Z., Zhang C., He D., Zhou Z., Li N., Yuan J. (2022). Inhibition of Galectin-3 Impairs Antifungal Immune Response in Fungal Keratitis. Dis. Markers.

[39] Azher T.N., Yin X.T., Stuart P.M. (2017). Understanding the Role of Chemokines and Cytokines in Experimental Models of Herpes Simplex Keratitis. J. Immunol. Res..

[40] Xiao Y., Yang J., Fu Z., He D., Li N., Yuan J. (2022). Galectin-3 Is a Crucial Immunological Disease Marker in Patients with Fungal Keratitis. Dis. Markers.

[41] Herre M., Cedervall J., Mackman N., Olsson A.K. (2023). Neutrophil extracellular traps in the pathology of cancer and other inflammatory diseases. Physiol. Rev..

[42] Mackey J.B.G., Coffelt S.B., Carlin L.M. (2019). Neutrophil Maturity in Cancer. Front. Immunol..

[43] Akinsipe T., Mohamedelhassan R., Akinpelu A., Pondugula S.R., Mistriotis P., Avila L.A., Suryawanshi A. (2024). Cellular interactions in tumor microenvironment during breast cancer progression: New frontiers and implications for novel therapeutics. Front. Immunol..

[44] Bronkhorst I.H., Jager M.J. (2013). Inflammation in uveal melanoma. Eye.

[45] Wolf J., Auw-Haedrich C., Schlecht A., Boneva S., Mittelviefhaus H., Lapp T., Agostini H., Reinhard T., Schlunck G., Lange C.A.K. (2020). Transcriptional characterization of conjunctival melanoma identifies the cellular tumor microenvironment and prognostic gene signatures. Sci. Rep..

[46] Diem S., Schmid S., Krapf M., Flatz L., Born D., Jochum W., Templeton A.J., Früh M. (2017). Neutrophil-to-Lymphocyte ratio (NLR) and Platelet-to-Lymphocyte ratio (PLR) as prognostic markers in patients with non-small cell lung cancer (NSCLC) treated with nivolumab. Lung Cancer.

[47] Nøst T.H., Alcala K., Urbarova I., Byrne K.S., Guida F., Sandanger T.M., Johansson M. (2021). Systemic inflammation markers and cancer incidence in the UK Biobank. Eur. J. Epidemiol..

[48] Templeton A.J., McNamara M.G., Šeruga B., Vera-Badillo F.E., Aneja P., Ocaña A., Leibowitz-Amit R., Sonpavde G., Knox J.J., Tran B. (2014). Prognostic role of neutrophil-to-lymphocyte ratio in solid tumors: A systematic review and meta-analysis. J. Natl. Cancer Inst..

[49] Cupp M.A., Cariolou M., Tzoulaki I., Aune D., Evangelou E., Berlanga-Taylor A.J. (2020). Neutrophil to lymphocyte ratio and cancer prognosis: An umbrella review of systematic reviews and meta-analyses of observational studies. BMC Med..

[50] Biousse V., Newman N.J. (2016). Diagnosis and clinical features of common optic neuropathies. Lancet Neurol..

[51] Cen L.P., Park K.K., So K.F. (2023). Optic nerve diseases and regeneration: How far are we from the promised land?. Clin. Exp. Ophthalmol..

[52] Kapoor D., Shukla D. (2023). Neutrophil extracellular traps and their possible implications in ocular herpes infection. Pathogens.

[53] Okruszko M.A., Szabłowski M., Zarzecki M., Michnowska-Kobylińska M., Lisowski Ł., Łapińska M., Stachurska Z., Szpakowicz A., Kamiński K.A., Konopińska J. (2024). Inflammation and Neurodegeneration in Glaucoma: Isolated Eye Disease or a Part of a Systemic Disorder?—Serum Proteomic Analysis. J. Inflamm. Res..

[54] Tang B., Li S., Han J., Cao W., Sun X. (2020). Associations between Blood Cell Profiles and Primary Open-Angle Glaucoma: A Retrospective Case-Control Study. Ophthalmic Res..

[55] Salvetat M.L., Pellegrini F., Spadea L., Salati C., Zeppieri M. (2023). Non-Arteritic Anterior Ischemic Optic Neuropathy (NA-AION): A Comprehensive Overview. Vision.

[56] Polat O., Yavaş G.F., İnan S., İnan Ü.Ü. (2015). Neutrophil-to-Lymphocyte Ratio as a Marker in Patients with Non-arteritic Anterior Ischemic Optic Neuropathy. Balk. Med. J..

[57] Gunes A., Yigit M., Tok L., Tok O. (2017). Neutrophil to Lymphocyte Ratio in Patients with Nonarteritic Anterior Ischemic Optic Neuropathy. Korean J. Ophthalmol..

[58] Bennett J.L. (2019). Optic Neuritis. Continuum.

[59] Balog B.M., Sonti A., Zigmond R.E. (2023). Neutrophil biology in injuries and diseases of the central and peripheral nervous systems. Prog. Neurobiol..

[60] Chakraborty S., Tabrizi Z., Bhatt N.N., Franciosa S.A., Bracko O. (2023). A Brief Overview of Neutrophils in Neurological Diseases. Biomolecules.

[61] Shafqat A., Noor Eddin A., Adi G., Al-Rimawi M., Abdul Rab S., Abu-Shaar M., Adi K., Alkattan K., Yaqinuddin A. (2023). Neutrophil extracellular traps in central nervous system pathologies: A mini review. Front. Med..

[62] Kurimoto T., Yin Y., Habboub G., Gilbert H.Y., Li Y., Nakao S., Hafezi-Moghadam A., Benowitz L.I. (2013). Neutrophils express oncomodulin and promote optic nerve regeneration. J. Neurosci..

[63] O’Koren E.G., Yu C., Klingeborn M., Wong A.Y.W., Prigge C.L., Mathew R., Kalnitsky J., Msallam R.A., Silvin A., Kay J.N. (2019). Microglial Function Is Distinct in Different Anatomical Locations during Retinal Homeostasis and Degeneration. Immunity.

[64] Calippe B., Augustin S., Beguier F., Charles-Messance H., Poupel L., Conart J.B., Hu S.J., Lavalette S., Fauvet A., Rayes J. (2017). Complement Factor H Inhibits CD47-Mediated Resolution of Inflammation. Immunity.

[65] Beguier F., Housset M., Roubeix C., Augustin S., Zagar Y., Nous C., Mathis T., Eandi C., Benchaboune M., Drame-Maigné A. (2022). The 10q26 Risk Haplotype of Age-Related Macular Degeneration Aggravates Subretinal Inflammation by Impairing Monocyte Elimination. Immunity.

[66] Bhutto I.A., McLeod D.S., Jing T., Sunness J.S., Seddon J.M., Lutty G.A. (2016). Increased choroidal mast cells and their degranulation in age-related macular degeneration. Br. J. Ophthalmol..

[67] Takeda A., Baffi J.Z., Kleinman M.E., Cho W.G., Nozaki M., Yamada K., Kaneko H., Albuquerque R.J., Dridi S., Saito K. (2009). CCR3 is a target for age-related macular degeneration diagnosis and therapy. Nature.

[68] Tsang S.H., Sharma T. (2018). Stargardt Disease. Adv. Exp. Med. Biol..

[69] Yogalingam G., Lee A.R., Mackenzie D.S., Maures T.J., Rafalko A., Prill H., Berguig G.Y., Hague C., Christianson T., Bell S.M. (2017). Cellular Uptake and Delivery of Myeloperoxidase to Lysosomes Promote Lipofuscin Degradation and Lysosomal Stress in Retinal Cells. J. Biol. Chem..

[70] Niazi S., Krogh Nielsen M., Subhi Y. (2019). Neutrophil-to-lymphocyte ratio in age-related macular degeneration: A systematic review and meta-analysis. Acta Ophthalmol..

[71] Ilhan N., Daglioglu M.C., Ilhan O., Coskun M., Tuzcu E.A., Kahraman H., Keskin U. (2015). Assessment of Neutrophil/Lymphocyte Ratio in Patients with Age-related Macular Degeneration. Ocul. Immunol. Inflamm..

[72] Krogh Nielsen M., Hector S.M., Allen K., Subhi Y., Sørensen T.L. (2017). Altered activation state of circulating neutrophils in patients with neovascular age-related macular degeneration. Immun. Ageing.

[73] Valapala M., Wilson C., Hose S., Bhutto I.A., Grebe R., Dong A., Greenbaum S., Gu L., Sengupta S., Cano M. (2014). Lysosomal-mediated waste clearance in retinal pigment epithelial cells is regulated by CRYBA1/βA3/A1-crystallin via V-ATPase-MTORC1 signaling. Autophagy.

[74] Ghosh S., Sharma R., Bammidi S., Koontz V., Nemani M., Yazdankhah M., Kedziora K.M., Wallace C.T., Yu-Wei C., Franks J. (2023). The AKT2/SIRT5/TFEB pathway as a potential therapeutic target in atrophic AMD. bioRxiv.

[75] Lavalette S., Raoul W., Houssier M., Camelo S., Levy O., Calippe B., Jonet L., Behar-Cohen F., Chemtob S., Guillonneau X. (2011). Interleukin-1β inhibition prevents choroidal neovascularization and does not exacerbate photoreceptor degeneration. Am. J. Pathol..

[76] Berchtold D., Priller J., Meisel C., Meisal A. (2020). Interaction of microglia with infiltrating immune cells in the different phases of stroke. Brain Pathol..

[77] Binet F., Cagnone G., Crespo-Garcia S., Hata M., Neault M., Dejda A., Wilson A.M., Buscarlet M., Mawambo G.T., Howard J.P. (2020). Neutrophil extracellular traps target senescent vasculature for tissue remodeling in retinopathy. Science.

[78] Ayoub T., Patel N. (2009). Age-related macular degeneration. J. R. Soc. Med..

[79] Handa J.T., Bowes-Rickman C., Dick A.D., Gorin M.B., Miller J.W., Toth C.A., Ueffing M., Zarbin M., Farrer L.A. (2019). A systems biology approach towards understanding and treating non-neovascular age-related macular degeneration. Nat. Commun..

[80] Kropp M., Golubnitschaja O., Mazurakova A., Koklesova L., Sargheini N., Vo T.K.S., de Clerck E., Polivka J., Potuznik P., Polivka J. (2023). Diabetic retinopathy as the leading cause of blindness and early predictor of cascading complications-risks and mitigation. EPMA J..

